# The impact of bariatric surgery and exercise on systemic immune inflammation index in patients with sarcopenia obesity

**DOI:** 10.1038/s41598-025-89806-3

**Published:** 2025-02-12

**Authors:** Cláudia Mendes, Manuel Carvalho, Jorge Bravo, Sandra Martins, Armando Raimundo

**Affiliations:** 1 Unidade Local Saúde Alentejo Central - Hospital Espírito Santo de Évora, EPE, Évora, Portugal; 2 CRI.COM - Centro Responsabilidade Integrada de Cirurgia da Obesidade e Metabólica, Évora, Portugal; 3 CHRC - Comprehensive Health Research Centre, Universidade de Évora, Évora, Portugal; 4 Departamento de Desporto e Saúde, Escola de Saúde e Desenvolvimento Humano, Universidade de Évora, Évora, Portugal; 5 CBIOS - Universidade Lusófona’s Research Center for Biosciences & Health Technologie, Lisboa, Portugal; 6 Research Center in Sports Sciences, Health and Human Development, CIDESD, Vila Real, Portugal; 7 Universidade Europeia, Lisboa, Portugal

**Keywords:** Bariatric surgery, Obesity, Systemic immune-inflammation index, Inflammation, Sarcopenia obesity, Diseases, Health care, Medical research, Risk factors, Musculoskeletal system, Biomarkers, Diagnostic markers, Predictive markers, Prognostic markers

## Abstract

The role of obesity in contributing to inflammation is an influential factor in the progression of obesity-associated medical issues. Metabolic and bariatric surgery has been proven as effective in obtaining weight loss and associated conditions remission. The Systemic Immune Inflammation Index (SII) was developed to offer more comprehensive data on inflammation and is presented as a prognostic indicator regarding many adverse conditions. The present study aimed to investigate the association between SII and bariatric surgery in patients with sarcopenic obesity and evaluate the eventual impact of exercise on SII. All participants were sarcopenic patients with obesity, underwent bariatric surgery - RYGP - and were randomized to participate in a structured physical exercise or to control group. The assessments were performed following standardized procedures, with the data evaluated during routine clinic follow-up at preoperative and 20-weeks postoperative after the exercise program. At baseline, before surgery, patients in both groups had similar anthropometrics, body composition, muscle strength variables and percentage of comorbidities. SII was also similar in both groups. To better understand the association of SII with the different variables, a Pearson correlation test was performed at baseline using SII. There was an inverse association of SII with BMC, handgrip strength and ASMM at baseline, which was maintained 5 months after surgery. At the end of the study, the combined results of the two groups showed that weight, BMI, % of body fat, muscle mass and muscle strength, the 30s sit-to-stand test and bone mineral density all decreased significantly as expected, along with the SII that also decreased significantly. The intervention group showed higher ASMM, handgrip strength, 30s Sit-to-stand test and 400-m walk test and bone mineral density when compared with the control group. However, SII showed no difference between both groups (*p* > 0.05). The results of the current research show a positive impact of bariatric surgery on weight and associated conditions control and a negative impact on muscle mass and function. SII responded very favorably to surgery with or without exercise, with a clear decrease in its score. Higher SII is associated with lower muscle mass and function, and this may be a reflex of the compromise that obesity causes on health, in this case, increasing systemic inflammation and decreasing muscle mass and function. The role of physical exercise in the management of surgical bariatric patients is still not clear. After surgery, the patients in the physical exercise program group had better results in muscle mass and function when compared to the patients in the control group (without exercise). However, there were no differences in SII score between the two groups, which may be interpreted as a lack of positive effect of physical exercise per se in the short-term on the systemic inflammatory condition present in obesity.

## Introduction

The World Health Organization (WHO) defines obesity as an abnormal or excessive fat accumulation that poses a risk to health^1^. Obesity not only causes serious economic costs but also increases the risk of several medical conditions, such as hypertension, diabetes, and obstructive sleep apnea^2^. The association between obesity and chronic low-grade inflammation, known as meta-inflammation, is well-documented. This chronic inflammation contributes to the progression of various diseases. Consequently, there is a growing interest in developing strategies to prevent the onset and progression of obesity-related diseases^2,3^.

Metabolic and bariatric surgery provide long-term effectiveness in weight loss and yields satisfactory results in the remission of conditions that are associated with cardiovascular risk and obesity^4–6^. The American Society of Metabolic and Bariatric Surgery (ASMBS) and the International Federation for the Surgery of Obesity and Metabolic Disorders (IFSO) recommend MBS in individuals with a body mass index (BMI) > 35 kg/m2, regardless of the presence and severity of comorbidities^7,8^.

The Systemic Immune-Inflammation Index (SII), a novel measure for inflammation, was created by Hu et al. in 2014^9^ and is a multi-marker index that provides a comprehensive assessment of the systemic immune-inflammatory response in the human body^10^. This index is a combination of independent white blood cells and platelets and is believed to reflect the interaction between thrombocytosis, inflammation, and immunity^11^ predicts worse prognosis for various medical conditions, disease recurrence and patient survival after surgery^10^. Studies show that the SII objectively reflects the inflammation-immunity balance in malignant tumor patients^12,13^ and is a prognostic indicator^14^. Elevated SII levels have been associated with worse prognoses for several medical conditions and higher mortality in patients with cancer and cardiovascular disease^15^. Some studies have suggested that SII serves as a marker of chronic inflammation^16^.

Sarcopenia, the age-related loss of skeletal muscle mass and function, has emerged as a significant public health concern in our aging global population^17^. This progressive condition not only impacts physical performance and quality of life but also increases the risk of adverse health outcomes, including falls, fractures, and mortality^18^. As researchers strive to understand the complex pathophysiology of sarcopenia, attention has been increasingly focused on the role of chronic low-grade inflammation, often referred to as inflammation, in its development and progression^19^.

While initially developed and validated in oncology settings, the potential utility of SII in age-related conditions like sarcopenia is now being explored. The relationship between inflammation and sarcopenia is multifaceted, involving complex interactions between pro-inflammatory cytokines, oxidative stress, and muscle protein metabolism^20^. Chronic inflammation has been implicated in promoting muscle catabolism, impairing muscle protein synthesis, and interfering with muscle regeneration processes. Given these connections, the SII may offer valuable insights into the inflammatory status of individuals at risk for or already experiencing sarcopenia^21^.

The complex interplay between physical activity and the immune system has also been a subject of increasing interest in recent years. As researchers continue to unravel the multifaceted effects of exercise on human health, attention has turned to various biomarkers that may provide insights into the body’s inflammatory and immune responses to physical exertion^22^. Initially developed in the context of cancer prognosis, the SII has since been explored in various other health conditions, including cardiovascular diseases and metabolic disorders. However, its potential role in exercise physiology and sports medicine remains relatively unexplored.

Exercise is known to induce acute and chronic changes in the immune system, with effects varying based on its intensity, duration, and type of physical activity^23^. Understanding these changes through easily accessible biomarkers like the SII could provide valuable insights into exercise-induced inflammation, recovery processes, and potential long-term health implications of different exercise regimens^24^.

In the present study, the purpose was to investigate the impact of bariatric surgery in the Systemic Immune Inflammation Index in Sarcopenic Obesity patients and to study the impact of exercise on the SII.

## Methods

### Study design and data collection

This randomized controlled trial (RCT) included patients with sarcopenia obesity who underwent gastric bypass (RYGB). The investigation is part of the EXPOBAR protocol, performed at a single center of Bariatric and Metabolic Surgery, involving the Hospital (ULSAC) and the University (ESDH-CHRC). The complete protocol has been described previously^25^.

The invitation to participate was made in the context of the preoperative evaluation, and participants who agreed to participate in the study were given the free and informed consent form previously approved by the University and Hospital Ethics Committee (Hospital Espírito Santo de Évora_Comissão de Ética - HESE_CE_1917/21). This research was presented following the Declaration of Helsinki and all experiments were performed following relevant guidelines and regulations. Informed consent was obtained from all subjects.

The participants were randomly assigned to either the intervention group (IG), which received a structured exercise program, or the control group (CG), which received standard care without additional exercise intervention. Exercise training began one month after surgery and was conducted three times per week for 16 weeks, for a maximum of 55 min per session.

The sociodemographic characteristics, perioperative, blood tests and body composition were assessed. The data was retrieved from the hospital’s electronic database. DEXA, handgrip test, 400-m walk test and 30s Sit-to-stand test, were evaluated in the Exercise and Health laboratory of the School of Health and Human Development of the University of Évora.

**Fig. 1:**
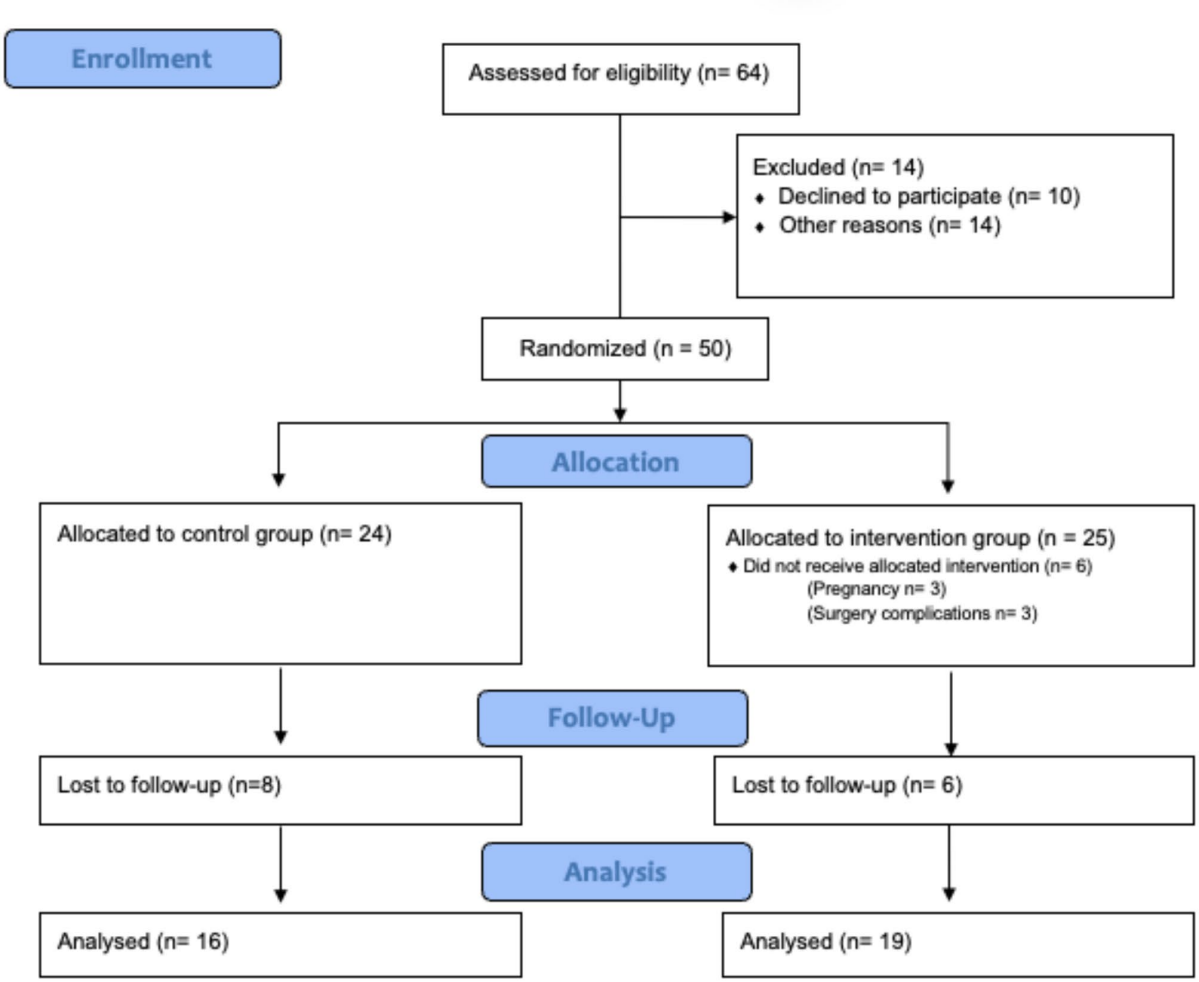
Consort flow diagram.

Researchers conducted all assessments without knowledge of the study’s goals or participants’ group assignments, reducing potential biases and safeguarding the data’s integrity. This study followed the CONSORT 2010 guidelines (Fig. 1)^26^.

### Eligibility criteria

Patients enrolled in the study were patients with an indication for bariatric surgery who also had a diagnosis of sarcopenia based on the criteria of the European Association for the Study of Obesity/European Society for Clinical Nutrition and Metabolism (EASO/ESPEN) and that agreed to participate in the study. Patients who reported problems with locomotion, neurological conditions that can affect balance or cognition, other previous bariatric surgery, or bariatric surgery complications were excluded.

### Intervention

Intervention Group: The intervention group participated in a structured exercise program designed to improve muscle strength, endurance, and overall physical function. A certified exercise physiologist supervised each session to ensure proper technique and safety. The program lasted 16 weeks, three times a week, for up to 55 min per session, starting one month after surgery. Each session began with 5 min of warm-up and ended with 10 min of cool-down^25,27,28^. The intervention was a progressive combined exercise program based on the FITT-VP (frequency, type, intensity, time, type, duration, volume, and progression) prescription^27,29^ as described in our previous paper^25,28^. The detailed combined exercise program is presented in Table 1.

Control Group: Participants in the control group received standard care, including regular health check-ups and nutritional counseling, but did not participate in any additional structured exercise program.

**Table 1:** Training periodization.

	F	I	T	T			V	*P*
	Frequency	Intensity	Time	Type			Volume	Progression
Training	*Warm-up*: 5 min on the treadmill − 50–60% FC reserve							
Phase 1 - Week 1–4 *Resistance*	3x/week	− 40–59% RHR − 10–12 Borg	35/39/43 min	ST	3/4/5 min		1 set-15-20 rep (1ª + 2ª week) 2 sets-12-15 rep (3ª + 4ª week)	Intensity Time
				AT	7/8/9 min			
				ST	3/4/5 min			
				AT	7/8/9 min			
Phase 2 - Week 5–10 *Hypertrophy*	3x/week	− 60–80% RHR − 12–14 Borg	45 min	ST	5 min		2 sets 12–15 rep	Intensity Time
				AT	10 min			
				ST	5 min			
				AT	10 min			
Phase 3 - Week 11–16 *Strength*	3x/week	− 70–89% RHR - > 14 Borg	55 min	ST	8 min		3 sets 10–12 rep	Intensity Time
				AT	12 min			
				ST	8 min			
				AT	12 min			
	*Cool-down*: up to 10 min - flexibility (myofascial release, mobility, static and dynamic stretching)							

ST: strength training; AT: aerobic training; min: minutes; rep: repetitions: RHR: reserve heart rate.

### Sample size and randomization

This study is a secondary analysis of the registered randomized controlled trial NCT05289219 at Clinicaltrials.gov^25^. The sample size was calculated by the G*power^30^. A total of 35 participants were enrolled in the study, with 19 in the IG and 16 in the CG, to enable the detection of a moderate estimated effect size (between-group differences) of at least 0.99 standard deviations in the outcome risk of sarcopenia^31,32^. Two-way independent sample t-tests were performed with an alpha error of α = 0.05 and a power of 1-β = 0.80.

Patients proposed for bariatric surgery (gastric bypass-RYGB) were randomly assigned at the time of proposal to usual care (CG) or usual care with an exercise program (IG). Patients were assigned to treatment groups using simple randomization with a random allocation rule, ensuring equal group sizes at the trial’s conclusion. The sequence generation utilized a random-number table.

### Primary outcome

The secondary outcome of the present study was to evaluate the impact of exercise on SII after bariatric surgery.

### Secondary outcome

The primary aim of this study was to investigate the association between Systemic Immune Inflammation Index and bariatric surgery in sarcopenic patients.

### Variables

*Anthropometry and body composition*: Anthropometric measurements of weight (in kilograms) and height (in centimeters) were taken, and the BMI was calculated. The participants’ body composition was assessed using Dual-energy X-ray absorptiometry (DEXA or DXA) with the Hologic QDR system from Hologic, Inc., based in Bedford, MA, USA. During the DEXA procedure, participants were required to fast and abstain from wearing any metal items or jewelry. Additionally, the study analyzed the total weight loss percentage (%TWL) by comparing participants’ initial and end of the study weights.

*Preoperative blood tests*: Preoperative blood tests were collected to analyze markers associated with obesity. These blood tests were performed both before surgery and after the exercise program. According to the hospital’s protocol, the first sample was taken in the week of preparation for surgery, and the second was obtained after the end of the exercise program.

*Systemic Immune Inflammation Index – SII*: Platelet (PLT) count, neutrophil (NEU) count and lymphocyte (LYN) count (expressed as ×10^3^ cells/µl) were measured by hematology analyzers and validated by a pathologist. The following formula was utilized to calculate SII= (PLT count × NEU count)/LYN count [13].

*Muscle strength*: To evaluate the muscle strength of the upper limbs, a handgrip strength test was conducted via manual pressure dynamometry (handgrip). The participants were instructed to stand with their elbows fully relaxed and straight. Each hand was tested twice, and the maximum grip strength value obtained was recorded as the muscle strength test value^33,34^. The muscle strength of the lower limbs was evaluated via the sit-to-stand test, in which participants were instructed to stand and sit for 30 s as many times as possible^35^. The timed chair stand test is a variation that counts how many times a patient can rise and sit in the chair over a 30-second interval^36,37^. Because the chair stand test evaluates both strength and endurance, it offers a reliable yet practical measure of strength but may be confounded by changes in weight after surgery.

*Muscle mass*: Muscle quantity or mass is evaluated by dual-energy X-ray absorptiometry (DEXA) because it is a common method for measuring skeletal muscle mass^38^. Skeletal muscle mass refers to the amount of muscle that is attached to the skeleton and helps in systemic movement and maintaining posture, which means that the sum of the muscle masses of the four limbs is defined as the appendicular skeletal muscle mass (ASMM)^39^. To calculate appendicular skeletal muscle mass (ASMM), we used the sum of the muscle masses of the upper and lower limbs (muscle mass of the arms [kg] + muscle mass of the legs [kg]). ASMM was divided by weight (meters) to diagnose sarcopenia (ASMM/weight)^40,41^. The ASMM score has been used to assess sarcopenic obesity^42^.

*Physical Performance*: The 400-m walk test was used to measure walking ability and endurance. The participants were asked to complete 20 laps of 20 m each as fast as possible and were allowed up to two rest stops during the test^43,44^. Low physical performance was considered when the test was not completed or when it took more than 6 min to complete^45^.

**Fig. 2:**
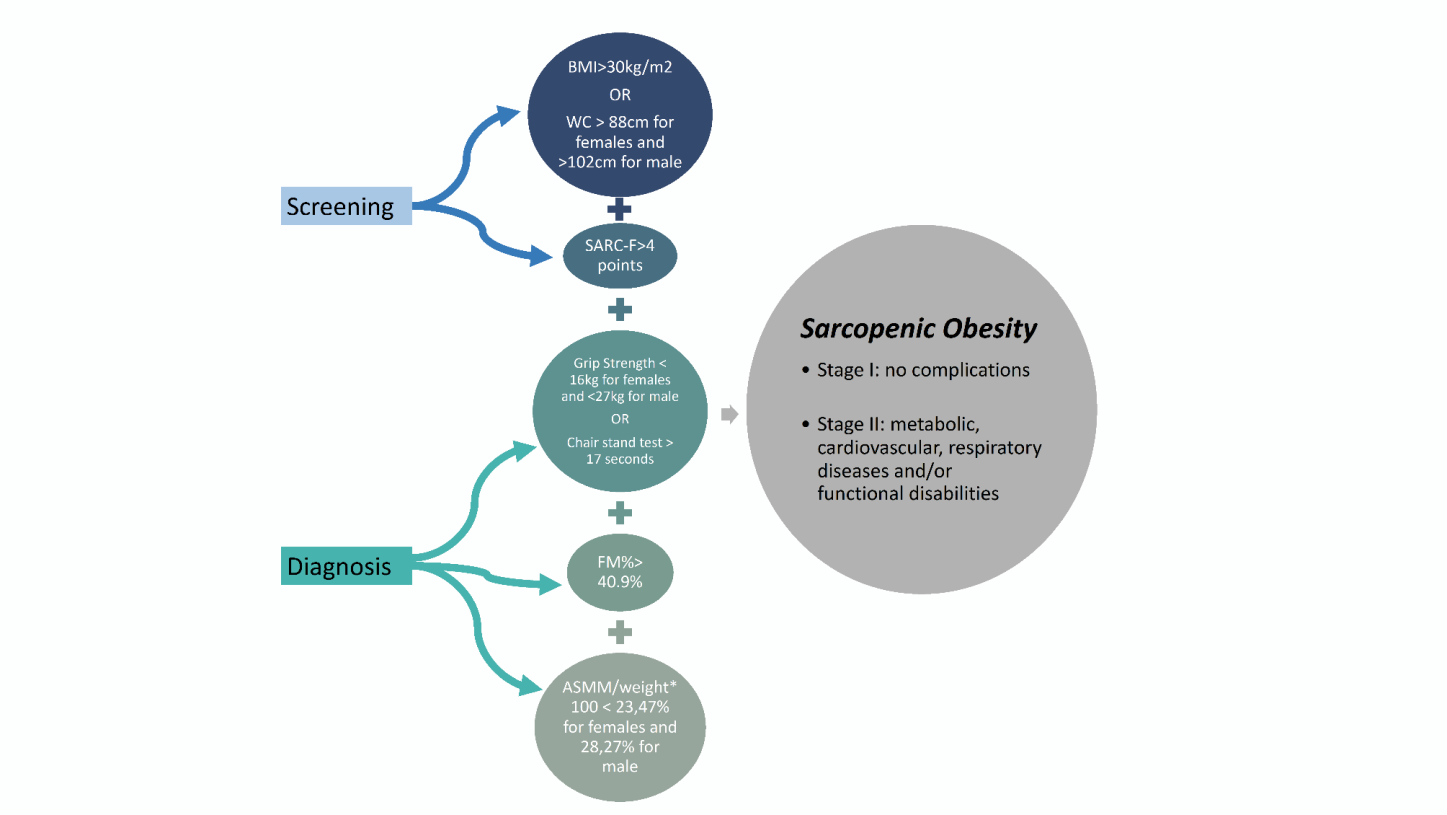
Algorithm based on the ESPEN-EASO criteria for sarcopenic obesity. ASMM: Appendicular Skeletal Muscle Mass, BMC: Body mineral content.

*Sarcopenic obesity*: Sarcopenia is diagnosed and considered severe when a high BMI or waist circumference combined with low muscle mass, low muscle strength and low physical performance are identified (Fig. 2). The first diagnostic criterion for sarcopenia is low muscle strength. Low muscle strength was defined as a handgrip strength of < 27 kg for males and < 16 kg for females^46^ and low muscle mass by DEXA based on ASMM/weight*100 (Cut-offs < 28.27% for M and < 23.47% for F)^41,47,48^.

### Statistical analysis

Parameters and outcomes were determined by statistical analysis using the computer software JAMOVI version 2.3.19. In descriptive statistics, mean ± standard deviation (SD) was used for parametric data, while median ± standard deviation (SD) was used for non-parametric data. Data normality was checked using the Shapiro-Wilk test, and group variances were examined with an independent t-test. Percentages were compared using the Chi-square test or the exact Fisher test. Dependent variables were compared using a two-way ANOVA and logistic regression analyses, considering group and two-time points before and after the exercise program.

## Results

A total of 35 patients were enrolled in this study. All patients met the criteria for sarcopenic obesity and the procedure of choice was always a RYGP. The preoperatory weight was 113 ± 17.3 kg, mean age was 46.9 ± 11.5 years and mean BMI was 43 ± 5.2. Diabetes was present in 17.1% of the patients, Dyslipidemia in 25.7%, and Hypertension in 68.6% of the participants. Baseline characteristics and clinical data of the participants are given in Tables 2 and 5.

**Table 2:** Sample baseline characteristics before surgery.

Variables (Mean ± SE)	Total *n* = 35	IG *n* = 19	CG *n* = 16	*p*-value
Age (years)	46.9 ± 11.5	43.7 ± 11.02	50.8 ± 11.29	0.071
Weight (Kg)	112.8 ± 17.3	118.3 ± 15.08	106.4 ± 17.99	0.041
BMI (kg/m^2^)	42.0 ± 5.16	43.2 ± 5.37	42.8 ± 5.05	0.825
Waist circumference (cm)	124.4 ± 10.9	125.2 ± 10.27	123.5 ± 11.97	0.662
Body fat (%)	47.0 ± 4.92	46.5 ± 5.92	47.6 ± 3.48	0.503
Total SMM mass (Kg)	56.69 ± 10.1	59.56 ± 8.67	53.46 ± 10.48	0.065
ASMM (Kg)	23.47 ± 4.80	24.86 ± 3.97	21.82 ± 5.28	0.061
ASMM/Weight (%)	20.8 ± 2.66	21.1 ± 2.95	20.4 ± 2.30	0.442
BMC (g)	2.47 ± 0.42	2.58 ± 0.39	2.32 ± 0.42	0.081
BMD (g/cm^2^)	1.18 ± 0.15	1.21 ± 0.16	1.14 ± 0.12	0.173
Total Body *T-score*	0.50 ± 1.39	0.55 ± 1.36	0.43 ± 1.47	0.812
Total Body *Z-score*	0.49 ± 1.15	0.41 ± 1.23	0.58 ± 1.07	0.647
Handgrip (Kg)	23.5 ± 9.42	28.02 ± 10.11	20.05 ± 6.48	0.010
30s Sit-to-stand test (n)	13.6 ± 3.34	14.68 ± 2.95	12.25 ± 3.38	0.029
400-m walk test (m)	6.98 ± 2.85	6.55 ± 2.85	7.49 ± 2.85	0.340
SII	504 ± 240	455 ± 136	563 ± 318	0.189
Glycemia (mg/dl)	99.3 ± 17.8	101 ± 22.3	97.6 ± 10.7	0.601
HbA1c (%)	4.83 ± 2.44	5.05 ± 2.58	4.56 ± 2.31	0.561
LDL (mg/dl)	173.8 ± 36.1	173 ± 40.2	175 ± 31.7	0.820
Triglycerides (mg/dl)	137.2 ± 56	132 ± 56.6	143.9 ± 56.4	0.524
HDL (mg/dl)	45.5 ± 15.5	46.4 ± 15.7	44.4 ± 15.7	0.820

BMI: Body Mass Index, SMM: Skeletal Muscle Mass, ASMM: Appendicular Skeletal Muscle Mass, ASMMI: Appendicular Skeletal Muscle Mass Index, HbA1c: Glycated Haemoglobin, SII: Systemic immune-inflamatory index, LDL: Colesterol, HDL: Colesterol.

The changes in the inflammatory indicators over time were examined in all patients, before surgery and at the end of the study (Table 3). A statistically significant decrease and large effect size was detected for anthropometric, body composition and osteoporosis parameters (*p* < 0.001; d > 0.8), but also in physical strength evaluated by handgrip (*p* < 0.001; d = 0.75) and sit-to-stand test (*p* = 0.011; d = 0.46). Overall, the 400-m walk test did not show differences after surgery, but the group who performed exercise had significant improvements (*p* = 0.002) when compared with the control group. Several obesity-associated diseases significantly improved, such as Diabetes (glycemia) and Dyslipidemia (LDL and triglycerides) parameters (*p* = 0.004; *p* = 0.026), but HbA1c did not have significant differences after surgery in any group.

**Table 3:** Main results before and after surgery.

Variables (Mean ± SE)	Surgery Effect			
	Before Surgery	After Surgery		
	Total sample *n* = 35	Total sample *n* = 35	*p*-value	Effect size (d)
Weight (Kg)	112.8 ± 17.3	80.0 ± 13.3	< 0.001	3.96
BMI (kg/m^2^)	42.0 ± 5.16	29.5 ± 4.27	< 0.001	4.32
Waist circumference (cm)	124.4 ± 10.9	97.5 ± 10.6	< 0.001	2.93
Body fat (%)	47.0 ± 4.92	38.6 ± 7.37	< 0.001	1.63
Total SMM mass (Kg)	56.69 ± 10.1	46.01 ± 9.33	< 0.001	2.80
ASMM (Kg)	23.47 ± 4.80	19.10 ± 4.18	< 0.001	3.18
ASMM/Weight (%)	20.8 ± 2.66	24.0 ± 3.88	< 0.001	-1.37
BMC (g)	2.47 ± 0.42	2.26 ± 0.41	< 0.001	0.83
BMD (g/cm^2^)	1.18 ± 0.15	1.12 ± 0.12	< 0.001	0.61
Total Body *T-score*	0.50 ± 1.39	0.27 ± 1.13	0.175	0.23
Total Body *Z-score*	0.49 ± 1.15	0.32 ± 0.87	0.126	0.27
Handgrip (Kg)	23.5 ± 9.42	21.1 ± 18.4	< 0.001	0.75
30s Sit-to-stand test (n)	13.6 ± 3.34	14.5 ± 3.36	0.011	0.46
400-m walk test (m)	6.98 ± 2.85	7.30 ± 2.85	0.359	-0.16
SII	504 ± 240	411 ± 191	0.024	0.401
Glycemia (mg/dl)	99.3 ± 17.8	89.4 ± 10.5	0.004	0.52
HbA1c (%)	4.83 ± 2.44	4.15 ± 2.63	0.201	0.22
LDL (mg/dl)	173.8 ± 36.1	157.8 ± 47.6	0.026	0.393
Triglycerides (mg/dl)	137.2 ± 56	107.6 ± 45.6	0.002	0.577
HDL (mg/dl)	45.5 ± 15.5	44.5 ± 16.5	0.731	0.059

BMI: Body Mass Index, BMC: Body mineral content, BMD: Body mineral density, SMM: Skeletal Muscle Mass, ASMM: Appendicular Skeletal Muscle Mass, ASMMI: Appendicular Skeletal Muscle Mass Index, HbA1c: Hemoglobin Glycate, SII: Systemic immune-inflammatory index, LDL: Cholesterol, HDL: Cholesterol.

*d* = Choen effect size; small = 0.2–0.49, medium = 0.5–0.79, large > 0.8.

A statistically significant decrease was detected in SII at the end of the study (Table 4) compared to the preoperative values (*p* = 0.024) with no differences between the exercise and control groups (*p* = 0.462). The IG also significantly improved muscle mass (*p* = 0.034), bone mineral content (*p* < 0.001), and physical function (*p* = 0.002) when compared with CG.

**Table 4:** Variation analysis after exercise.

Variables	Surgery + Exercise Effect			
(Mean ± SE)	IG *n* = 19	CG *n* = 16	*p*-value	Effect size (d)
Weight (Kg)	-20.1 ± 9.18	-16.9 ± 4.05	0.198	0.902
BMI (kg/m^2^)	-7.33 ± 3.28	-6,93 ± 2.10	0.681	-0.14
Waist circumference (cm)	-14.9 ± 5.99	-12.4 ± 9.44	0.345	-0.32
Total Weight Loss (%)	16.7 ± 6.36	16.3 ± 4.93	0.841	0.07
Body fat (%)	-7.55 ± 4.22	-5.09 ± 5.02	0.002	-1.13
Total SMM mass (Kg)	-4.97 ± 3.90	-3.41 ± 2.89	0.196	-0.45
ASMM (Kg)	-18.3 ± 12.7	-22.7 ± 11.4	0.034	3.18
ASMM/Weight (%)	3.07 ± 2.66	1.89 ± 1.78	0.141	0.51
BMC (g)	0.12 ± 0.01	0.05 ± 0.03	< 0.001	0.83
BMD (g/cm^2^)	0.06 ± 0.02	1.09 ± 0.07	0.114	0.61
Total Body *T-score*	0.03 ± 0.22	-0.32 ± 0.23	0.069	0.23
Total Body *Z-score*	0.01 ± 0.11	0.39 ± 0.32	0.451	0.27
Handgrip (Kg)	2.39 ± 5.23	-1.74 ± 3.66	0.002	0.75
30s Sit-to-stand test (n)	1.68 ± 2.06	0.38 ± 1.45	0.002	0.46
400-m walk test (min)	-1.18 ± 1.56	0.06 ± 1.21	0.002	-0.16
SII	-163 ± 56	-126 ± 115	0.462	0.401
Glycemia (mg/dl)	-12.9 ± 14.6	-7.7 ± 3.5	0.436	0.52
HbA1c (%)	-0.58 ± 0.06	-0.79 ± 0.66	0.441	0.22
LDL (mg/dl)	-0.11 ± 0.64	-22 ± 21.7	0.556	0.393
Triglycerides (mg/dl)	-0.27 ± 0.05	-0.45 ± 0.03	0.292	0.577
HDL (mg/dl)	-1.2 ± 0.22	-0.1 ± 0.05	0.957	0.059

The remission rates of various conditions (Diabetes, Hypertension, Dyslipidemia, and OASA) 5 months after surgery, comparing the intervention group (IG) with the control group (CG), are present in Table 5. It also examines the effects of surgery and surgery + exercise on these conditions.

**Table 5:** Associated obesity disease remission.

Variables	Remission 5-months after surgery						Surgery Effect	Surgery + Exercise Effect
	Before Surgery			After Surgery				
	IG *n* = 19	CG *n* = 16	*p*-value	IG *n* = 19	CG *n* = 16	*p*-value	*p*-value	*p*-value
Diabetes	5.7%	11,4%	0.271	2.9%	2.9%	0.904	0.046	0.317
Hypertension	34.3%	34.3%	0.467	5.7%	8.6%	0.503	< 0.001	0.002
Dyslipidemia	5.7%	20%	0.025	0%	8.6%	0.050	0.014	0.163
OASA	5.7%	20%	0.025	0%	8.6%	0.050	0.014	0.163

OASA: obstructive sleep apnea.

Table 6 presents data on the relationship between the SII and various body composition and physical function measures at baseline (E0) and after 5 months (E1). The columns provide information on each variable’s correlation coefficient (r2), p-value, and 95% confidence interval (CI).

A significant negative correlation between SII and BMC (***r***^***2***^ = − 0.373; *p* = 0.027; CI: -0.628; -0.045) and with *t-score* (***r***^***2***^ = − 0.447; *p* = 0.007; CI: -0.679; -0.133) at baseline. Five months after RYGB the negative correlation is with handgrip (***r***^***2***^ = − 0.367; *p* = 0.030; CI: -0.039; -0.624), ASMM (***r***^***2***^ = − 0.397; *p* = 0.018; CI: -0.645; -0.074) and ASMM/Weight (***r***^***2***^ = − 0.557; *p* < 0.001; CI: -0.751; -0.274), the EASO/ESPEN parameter to diagnose sarcopenia.

**Table 6:** Linear regression analysis after surgery and exercise based on SII.

Variables	Systemic immune-inflammatory index - SII					
	Before Surgery			After Surgery + Exercise		
	*r* ^2^	*p*-value	CI 95%	*r* ^2^	*p*-value	CI 95%
BMC (g)	-0.373	0.027	− 0.628; -0.045	-0.278	0.106	-0.559; 0.061
Body fat (%)	0.072	0.680	-0.664; -0.107	0.232	0.179	-0.109; 0.525
Handgrip (Kg)	-0.322	0.060	-0.591; 0.013	-0.367	0.030	-0.039; -0.624
ASMM (Kg)	-0.313	0.067	-0.585; 0.023	-0.397	0.018	-0.645; -0.074
ASMM/Weight (%)	-0.251	0.147	-0.539; 0.090	-0.557	< 0.001	-0.751; -0.274
Total Body *T-score*	-0.447	0.007	-0.679; -0.133	-0.254	0.140	-0.542; 0.086

ASMM: Appendicular Skeletal Muscle Mass, BMC: Body mineral content.

## Discussion

This study evaluated the effects of Roux-en-Y gastric bypass (RYGB) surgery and subsequent exercise interventions on SII in a cohort of 35 patients diagnosed with sarcopenic obesity.

Inflammatory conditions can be assessed by the SSI. This index includes neutrophils, lymphocytes, and platelet count in a blood sample. It is a simple, efficient, and low-cost test. Other studies have shown that it has a predictor value in tumors, cardiovascular disease, hepatics steatosis, osteoporosis^49^, diabetes and other conditions. Higher levels of SSI are associated with worse prognosis and increasing mortality^50^.

Our baseline characteristics reveal a population with severe obesity, sarcopenic obesity and a high prevalence of related comorbidities, setting the stage for the assessment of the potential benefits of RYGB surgery. Preoperatively, higher SII is associated with lower muscle mass and function, and this may be a reflex of the compromise that obesity causes on health, in this case, simultaneously increasing systemic inflammation and affecting muscle mass and function.

After surgery, our results show a favorable impact of bariatric surgery on weight and associated conditions control and a negative impact on muscle mass and function. SII responds very favorably to surgery with or without exercise, with a clear decrease in its score.

The study shows significant improvements in anthropometric and body composition parameters after surgery. The reductions in weight, BMI, and body fat percentage were statistically significant with large effect sizes. These findings are consistent with the expected outcomes of bariatric surgery, which typically results in substantial weight loss and improved body composition^51,52^.

Lin Shi et al., studied the relationship between SSI and muscle mass. They concluded that the increased SII levels were associated with an increased risk of low muscle mass in a large population. This association is present in the patients in our study before surgery. All have sarcopenic obesity with low muscle mass assessed by ASMM/weight, and the mean SII is high. However, after surgery, there is a decrease in SII but also in muscle mass. If we extrapolate the results from Lin we should have the inverse result, but we can reason that the bariatric surgery influence on weight loss and muscle mass loss is greater than the protective effect that can result from decreasing SII^53^.

There were significant improvements in Diabetes (glycemia) and Dyslipidemia (LDL and triglycerides) postoperatively. However, HbA1c levels did not show significant differences. The remission rates for Diabetes, Hypertension, Dyslipidemia, and OSAS also improved significantly post-surgery, highlighting the surgery’s efficacy in managing obesity-related diseases^4,6,54^. However, there were no differences between the intervention and the control groups.

Nevertheless, the role of physical exercise in the management of surgical bariatric patients is still not clear. Physical strength, measured by handgrip and sit-to-stand tests, improved postoperatively in the intervention group but not in the control group, and the difference at the end of the study was significant. This indicates that, while RYGB surgery alone may not improve strength, combining it with exercise leads to better functional outcomes. After surgery the patients in the physical exercise program group had better results in muscle mass and strength when compared to the patients in the control group (without exercise).

The SII significantly decreased when measured five months after surgery, suggesting reduced systemic inflammation. The lack of significant differences in the exercise group compared to the control group could imply that surgery or weight loss plays a more significant role in reducing inflammation than exercise^11,53^. However, after surgery with exercise, the group that exercised had better results, and linear regression shows that more significant reductions in inflammation are associated with better results in muscle mass (ASMM and ASMM/weight) and strength, highlighting the interconnectedness of the inflammatory status and physical health in sarcopenic obesity.

However, there were no significative differences in SII score between the two groups, which may be interpreted as a lack of positive effect of physical exercise on the systemic inflammatory condition in obesity.

## Conclusion

This study underscores the multifaceted benefits of RYGB surgery in patients with sarcopenic obesity. RYGB showed effects that were considered positive on inflammatory markers obtained from routine blood tests. Significant improvements were observed in weight, body composition, comorbidities, and inflammatory markers. The addition of exercise further enhanced physical function. The correlations between SII and various health metrics suggest that reducing systemic inflammation through surgery could play a critical role in improving muscle mass and especially physical strength. These findings support the integrated approach of combining surgical and exercise interventions to optimize health outcomes in patients with sarcopenic obesity.

## Data Availability

Data availability Statements: This is a research article and all data generated or analyzed during this study are available. All inquiries should be directed to correspondence author.

## Abbreviations

WHO: World Health Organization
ASMBS: American Society of Metabolic and Bariatric Surgery
IFSO: International Federation for the Surgery of Obesity and Metabolic Disorders
BMI: Body Mass Index
SII: Systemic Immune-Inflammation Index
FITT-VP: frequency, type, intensity, time, duration, volume, and progression
ACSM: American College of Sports Medicine
RYGB: Roux en Y Gastric Bypass
DEXA: Dual-energy X-ray absorptiometry
TWL: total weight loss percentage
PLT: Platelet
NEU: neutrophil
LYN: lymphocyte
WC: waist circumference
SD: standard deviation
SMM: Skeletal Muscle Mass
ASMM: Appendicular Skeletal Muscle Mass
ASMMI: Appendicular Skeletal Muscle Mass Index
HbA1c: Glycated Haemoglobin
ESPEN: European Society for Clinical Nutrition and Metabolism
EASO: European Association for the Study of Obesity
FNIH: Foundation for the National Institutes of Health
